# *Lawsonella clevelandensis*, a case series of vascular graft infections caused by a rare pathogen

**DOI:** 10.1016/j.idcr.2023.e01735

**Published:** 2023-03-06

**Authors:** Shirin I. Nour, Ryan B. Khodadadi, Audrey N. Schuetz, Robin Patel, Omar M. Abu Saleh

**Affiliations:** a Department of Medicine, Mayo Clinic, Rochester, MN, USA; b Division of Public Health, Infectious Diseases, and Occupational Medicine, Mayo Clinic, Rochester, MN, USA; c Division of Clinical Microbiology, Department of Laboratory Medicine and Pathology, Mayo Clinic, Rochester, MN, USA

**Keywords:** *Lawsonella clevelandensis*, Vascular graft infection, Rare pathogen

## Abstract

*Lawsonella clevelandensis* is a fastidious Gram-positive, partially acid-fast, anaerobic, catalase positive bacterium that has been reported to be a rare cause of abdominal, breast, spinal, and liver abscesses. Here, three *L. clevelandensis* vascular graft infections (VGIs) and cardiac infections are reported.

## Introduction

*Lawsonella clevelandensis* is a fastidious Gram-positive, partially acid-fast, anaerobic bacterium first described in 2013 as a pathogen in a series of monomicrobial abscesses leading to the description of a new Corynebacterineae suborder [1], [2], [3].

The diagnosis of *L. clevelandensis* is challenging given the limited ability to isolate this organism by routine anaerobic culture [4]. As a result, molecular diagnostic methods for identification such as 16S ribosomal RNA (rRNA) polymerase chain reaction (PCR)/sequencing have often been used to detect *L. clevelandensis* [3].

This organism remains a rare cause of infection and is part of the human skin and nasal microbiomes [5], [6], [7]. Recently, the first vascular graft infection (VGI) due to *L. clevelandensis* was described with pathogen identification via 16S rRNA PCR/sequencing at our institution [6]. We sought to review our institutional experience with *L. clevelandensis* infection, highlighting clinical manifestations and diagnosis, alongside treatment and subsequent outcomes related to VGI.

## Methods

Following Mayo Clinic Institutional Review Board (IRB) approval and research participation consent form patients, a retrospective review of adult patients (≥ 18 years of age) evaluated at Mayo Clinic in Rochester, Minnesota, with microbiologic identification of *L. clevelandensis* via 16S rRNA PCR/sequencing was conducted. Five patients were identified; three of the five had confirmed VGI attributed to this pathogen. Abstracted data included patient demographics, medical history, clinical course, and outcomes.

## Case 1(previously published)

A 65-year-old male with a history of infrarenal abdominal aortic aneurysm (AAA) with endovascular aneurysm repair with aorto-bi-iliac graft and L3–L5 spinal fusion (2016) presented in July 2018 with periumbilical abdominal pain and back pain with associated with chills/night sweats, and 30-pounds of unintentional weight loss. Computerized tomography (CT) of the abdomen/pelvis (A/P) revealed a left sided para-aortic psoas fluid collection prompting CT-guided aspiration resulting in negative bacterial (aerobic and anaerobic) cultures on blood sheep agar and anaerobic blood agar at 14 days, as well as negative fungal and mycobacterial cultures at 24 and 42 days.

He continued to experience low back and groin pain prompting positron emission tomography (PET)-CT revealing bilateral fluorodeoxyglucose (FDG)-avid psoas abscesses, inflammation within the AAA wall, and FDG uptake within the common iliac portion of the vascular graft, suggestive of infection. Laboratory results were notable for an erythrocyte sedimentation rate (ESR) of 100 mm/h (2–20 mm/h), C-reactive protein (CRP) of 132 mg/L (< 8 mg/L), leukocytosis to 17.5 × 10^9^/L (3.4–9.6 × 10^9^/L) and thrombocytosis of 758 × 10^9^/L (135–317 × 10^9^/L). Blood cultures were negative at 5 days. No antibiotics were given.

Approximately 60 days from original presentation, he underwent explantation of the endograft followed by replacement with a cryopreserved aortoiliac graft from the juxtarenal aorta to the right external iliac and left distal common iliac artery in addition to drainage and debridement of bilateral psoas abscesses. Operative aerobic and anaerobic bacterial culture at 14 days on blood sheep agar and anaerobic blood agar as well as fungal and mycobacterial cultures at 24 and 42 days respectively were negative. However, 16S rRNA gene PCR/sequencing on AAA sac fluid and aortic tissue detected *L. clevelandensis*. He was treated with vancomycin, cefepime, and doxycycline post-operatively and completed a 6-week course of intravenous (IV) vancomycin and oral (PO) doxycycline. He was continued on PO doxycycline and cefadroxil for 3 months followed by chronic suppressive doxycycline therapy.

## Case 2

A 65-year-old male with medical history of AAA status post endovascular stenting repair 3 months prior presented to the emergency department (ED) with progressive low back pain and fevers. At the time of endovascular stenting, the patient had leukocytosis and elevated inflammatory markers and was treated with 5 weeks of IV daptomycin post-operatively. In the ED, CT A/P showed a prominent soft tissue density surrounding the posterolateral wall of the aneurysm sac extending proximally. Magnetic resonance imaging (MRI) revealed an enhancing lesion involving the L3–L4 inferior endplates consistent with perivascular abscess and spinal osteomyelitis. He underwent CT-guided aspiration of the paraspinal and retroperitoneal abscesses and was started on IV daptomycin and ertapenem. One month later, he underwent lumbar spine interbody fusion with hardware placement. Post-operatively, he was treated with cefepime, metronidazole, and daptomycin for 3 weeks, followed by doxycycline suppression for 7 months.

Seven months later, repeat CT A/P revealed progression of right retroperitoneal fluid collections prompting drain placement and addition of ciprofloxacin to doxycycline. Repeat MRI one month later revealed a multiloculated abscess at the site of the prior fluid collection, not amenable to drainage leading to rehospitalization.

Evaluation revealed leukocytosis (13.1 × 10^9^/L), thrombocytosis (369 × 10^9^/L), elevated ESR (48 mm/h) and CRP (15.1 mg/L). Peripheral blood cultures were negative at 5 days. PET-CT revealed FDG-avid L3–L4 osteomyelitis with extension into the adjacent bilateral psoas muscles with a sinus tract from the left psoas muscle to the left flank subcutaneous tissues and intense uptake around the superior aspect of the aortic endograft with less intense uptake inferiorly throughout the remainder of the graft. The proximal iliac vessels were also involved. Together, these findings were consistent with VGI. He was initiated on cefepime, metronidazole, and daptomycin.

Following removal of the endovascular aneurysm stent, excision of the infrarenal aorta, debridement of bilateral psoas abscesses, and removal of anterior infected spinal hardware, he underwent aortoiliac reconstruction from the juxtarenal aorta to the left external iliac artery and right common artery with cryopreserved aortoiliac graft and omental graft coverage. Aerobic and anaerobic bacterial cultures on blood sheep and anaerobic blood agar at 14 days as well as fungal and mycobacterial cultures at 24 and 42 days from operative specimens were negative in addition to peripheral blood cultures which were negative at 5 days. However, 16S rRNA gene PCR/sequencing of aortic tissue returned positive for *L. clevelandensis* DNA. He was subsequently treated with IV ertapenem for 6 weeks and transitioned to lifelong PO amoxicillin-clavulanate suppression.

## Case 3

**Fig. 1:**
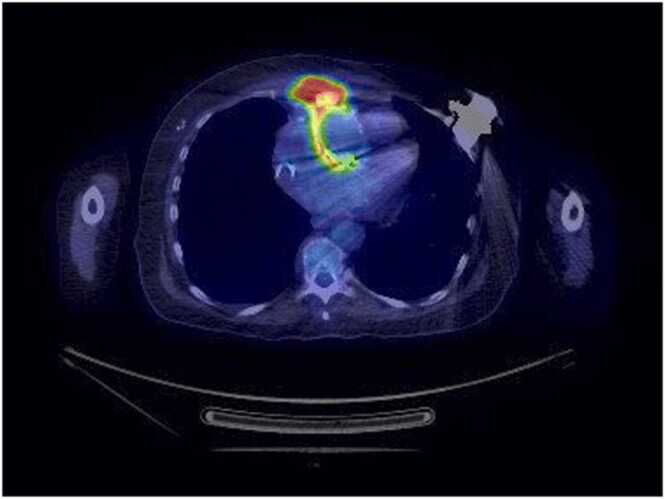
**PET-CT image of the chest.** PET-CT image demonstrating FDG uptake within the parasternal soft tissues with extension to the aortic root.

A 65-year-old female with a history of mechanical aortic valve (AV) replacement and aortic annulus patch augmentation (1999) was admitted for dyspnea, fatigue, and concern for infective endocarditis (IE). Her medical history included prior infection of sternotomy wires with skin breakdown and recurrent chest wall infections (2011) resulting in bilateral pectoral flap reconstruction (2017) and ventricular tachycardia with placement of a biventricular implantable cardiac defibrillator. Initial evaluation revealed negative bacterial blood cultures at 7 days. Transthoracic echocardiogram (TTE) revealed periprosthetic regurgitation and PET-CT demonstrated FDG uptake within the parasternal soft tissues with extension to the aortic root (Fig. 1). She was treated with 4 weeks of IV ceftriaxone and vancomycin prior to cardiac device removal followed by St. Jude AV replacement with a Hemashield patch, tricuspid valve repair, and debridement of the ascending aorta and root with graft and valve conduit placement. Intraoperatively, a large amount of purulent material under the pectoral flap and grossly infected AV with several areas of dehiscence were noted. Aerobic and anerobic bacterial culture on blood sheep agar and anaerobic blood agar were negative at 14 days of incubation in addition to negative fungal and mycobacterial cultures held for 24 and 42 days but AV tissue 16 S rRNA gene PCR/sequencing was positive for *L. clevelandensis*, prompting treatment with IV ceftriaxone and vancomycin. Her postoperative course was complicated by multiple debridement procedures and washouts. Her condition continued to decline, and she died after 13 days following a family decision to discontinue intensive care.

## Discussion

We highlight 3 cases of culture-negative, monomicrobial VGI due to *L. clevelandensis* diagnosed by 16S rRNA gene PCR/sequencing on surgically-collected fluids/tissues.

VGI is a serious complication of reconstructive vascular surgery associated with substantial mortality [8], [9]. Diagnosis is based on clinical, radiographic, and laboratory findings. The microbiology of VGI varies depending on the location of vascular graft reconstruction and mechanism of invasion, identification of a causative organism(s) being critical for selection of pathogen-directed therapy [6], [9].

While the etiology of infection in each case is uncertain, *L. clevelandensis* has been detected in human microbiome studies of skin and Intestinal microbiota. Contamination from skin microbiota or contiguous spread from an enteric source could be the mechanisms for VGI [3], [6].

In Cases 1 and 3, the onset of symptoms was indolent, occurring years after index surgery. In Case 2, the patient developed symptoms 3 months after graft surgery (Table 1). All patients required surgical intervention for source control and received induction antimicrobial therapy administered for 6 weeks post-operatively in Cases 1 and 2, followed by suppressive antibiotics.

**Table 1:** *Lawsonella clevelandensis* vascular infection cases.

Case	Age, Sex	Comorbidities	Microbiologic diagnosis	Culture data	Antimicrobial therapy	Outcome
**1**^a^	65, Male	AAA repair with graft, umbilical hernia s/p repair, coronary artery disease s/p CABG	16S rRNA gene sequencing	Negative aerobic and anaerobic peripheral blood cultures	IV Vancomycin and PO doxycycline for 6 weeks followed PO doxycycline and cefadroxil for 3 months then lifeline PO doxycycline suppression	Resolution of infection
**2**	65, Male	Hyperlipidemia, tobacco dependance	16S rRNA gene sequencing	Negative aerobic and anaerobic peripheral blood cultures	Daptomycin, ertapenem, cefepime, and metronidazole. After graft removal and replacement, patient was transitioned to ertapenem for 6 weeks, followed by lifelong amoxicillin-clavulanate suppressive therapy	Resolution of infection
**3**	65, Female	Bicuspid aortic valve s/p replacement with St. Jude AV with Hemashield patch aortic root enlargement, complete heart block and ventricular tachycardia s/p dual chamber ICD, and recurrent sternal wound infections	16S rRNA gene sequencing	Negative aerobic and anaerobic peripheral blood cultures	IV ceftriaxone and vancomycin for 4 weeks followed by post-operative IV vancomycin and ceftriaxone	Death
**4**^b^	70, Male	Hypertension, AAA, asthma, transient ischemic attacks, and infected native aortic aneurysm	16S rRNA gene sequencing	Negative aerobic and anaerobic cultures of intra-abdominal fluid aspirate	Teicoplanin and metronidazole for 9 days then transitioned to PO amoxicillin-clavulanate after surgical intervention	Death one month after surgical intervention

^a^Abbreviations: AAA, abdominal aortic aneurysm; S/P, status post; CABG, coronary artery bypass graft surgery; IV, intravenous; PO, oral; AV, aortic valve; ICD, implantable cardiac defibrillator.

a Case 1 data from Ref. [6].

b Case 4 not included in text, data from Ref. [10].

There is one reported case of native AAA *L. clevelandensis* infection (Table 1). To our knowledge, this report is the first case series highlighting a possible association of *L. clevelandensis* with VGI [10]. With the report of these cases of *L. clevelandensis* infection, VGI accounts for 3 of a total of 10 reported cases, making it the most common type of infection attributed to this species which may have received limited recognition as a culprit of VGI given the difficulties in cultivating it in culture. In all cases in this report, operative anaerobic cultures held for at least 14 days failed to recover *L. clevelandensis* and this pathogen was only detected by 16S rRNA PCR/sequencing from surgical specimens. Prior successful culture has been reported under strict anaerobic conditions, notably on Columbia sheep-blood agar [3]. Additional considerations for unsuccessful isolation on anaerobic culture in this series could include pre-operative antimicrobial exposure such as in Cases 2 and 3. Fortunately, the availability of 16S rRNA PCR/sequencing and other deep sequencing methods have made the diagnosis of such rare pathogens possible [11].

Data remain limited regarding treatment for *L. clevelandensis*. Prior studies demonstrate low minimal inhibitory concentrations to multiple antibiotic classes, including fluoroquinolones, beta-lactams, macrolides, and penicillins [3]. Additional studies will be needed to further define typical susceptibility patterns of this pathogen.

In summary, this study shows the clinical value of 16S rRNA gene PCR/sequencing in diagnosing the hitherto unrecognized clinical entity of *L. clevelandensis* VGI.

## CRediT authorship contribution statement

S.I.N. and R.B.K. contributed equally to the conception, preparation, and review of this manuscript. A.N.S., R.P., and O.A.S. contributed to the conception, design, preparation, and manuscript review. S.I.N., R.B.K, A.N.S., and O.A.S. have no conflicts to declare.

## Ethical approval

Following Mayo Clinic Institutional Review Board (IRB) approval and research participation consent form patients, a retrospective review of adult patients (≥ 18 years of age) evaluated at Mayo Clinic in Rochester, Minnesota, with microbiologic identification of *L. clevelandensis* via 16S rRNA PCR/sequencing was conducted. Five patients were identified; three of the five had confirmed VGI attributed to this pathogen. Abstracted data included patient demographics, medical history, clinical course, and outcomes.

## Consent

Patients included in this study have provided research authorization for the confidential clinical use of information to Mayo Clinic.

## Funding

No funding sources to declare for this work.

## Potential conflicts of interest

Dr. Patel reports grants from ContraFect, TenNor Therapeutics Limited, and BioFire, and Adaptive Phage Therapeutics (paid to institution). Dr. Patel is a consultant to Curetis, PathoQuest, Selux Diagnostics, 1928 Diagnostics, PhAST, Torus Biosystems, Day Zero Diagnostics, Mammoth Biosciences, HealthTrackRx, and Qvella; monies are paid to Mayo Clinic. Mayo Clinic and Dr. Patel have a relationship with Pathogenomix. Dr. Patel has research supported by Adaptive Phage Therapeutics. Mayo Clinic has a royalty-bearing know-how agreement and equity in Adaptive Phage Therapeutics. Dr. Patel is also a consultant to Netflix, Abbott Laboratories, and CARB-X. In addition, Dr. Patel has a patent on Bordetella pertussis/parapertussis PCR issued, a patent on a device/method for sonication with royalties paid by Samsung to Mayo Clinic, and a patent on an anti-biofilm substance issued. Dr. Patel receives honoraria from the NBME, Up-to-Date and the Infectious Diseases Board Review Course. Dr. Patel reports travel reimbursement to author from ASM and a volunteer role on the Governance Committee of ASM.
